# Weathering increases the acute toxicity of plastic pellets leachates to sea-urchin larvae—a case study with environmental samples

**DOI:** 10.1038/s41598-024-60886-x

**Published:** 2024-05-23

**Authors:** Michele Ferrari, Filipe Laranjeiro, Marta Sugrañes, Jordi Oliva, Ricardo Beiras

**Affiliations:** 1 ECIMAT, Centro de Investigación Mariña (CIM), Universidade de Vigo, 36331 Vigo, Galicia, Spain; 2 Associació Good Karma Projects, Manila 49 Àtic 2, 08034 Barcelona, Spain

**Keywords:** Microplastic, Leachates, Polyethylene, Polypropylene, Image processing, Environmental impact, Marine biology

## Abstract

Microplastics, particles under 5 mm, pervade aquatic environments, notably in Tarragona’s coastal region (NE Iberian Peninsula), hosting a major plastic production complex. To investigate weathering and yellowness impact on plastic pellets toxicity, sea-urchin embryo tests were conducted with pellets from three locations—near the source and at increasing distances. Strikingly, distant samples showed toxicity to invertebrate early stages, contrasting with innocuous results near the production site. Follow-up experiments highlighted the significance of weathering and yellowing in elevated pellet toxicity, with more weathered and colored pellets exhibiting toxicity. This research underscores the overlooked realm of plastic leachate impact on marine organisms while proposes that prolonged exposure of plastic pellets in the environment may lead to toxicity. Despite shedding light on potential chemical sorption as a toxicity source, further investigations are imperative to comprehend weathering, yellowing, and chemical accumulation in plastic particles.

## Introduction

Microplastics (MP), plastic particles or fragments smaller than 5 mm, have become widespread along the aquatic environments worldwide, posing a serious environmental problem due to their persistence, durability and toxic potential^1^. MPs are commonly classified as primary, intentionally produced within this size range, and secondary, derived from the degradation of larger plastics fragments, such as meso- and macro-plastics^2^.

Plastic pellets, typically found within primary MPs, are raw materials used to manufacture large scale plastic products, thus making them a common item found on sandy shores all over the world^3,4^. These items can be released into the environment during manufacturing and transport or as a result of accidental spills during marine and terrestrial shipping^5^. For instance, industrial pellets are listed as the main source of MPs to the marine environment in Spain^6^. Therefore, numerous studies have been conducted to understand their distribution in marine environments, the potential mechanical impacts on marine organisms and consequently their harmful effects on marine organisms (e.g. Ref^7^). Plastic materials may contain “plastic additives” used to improve their performance, such as phthalates (PAEs), organophosphate esters (OPEs) or bisphenols (BPs)^8^. Besides that, there is an increased interest on the accumulation of hydrophobic chemicals by the pellets. Given their large surface:volume ratio, microplastics have a high ability to absorb hydrophobic organic contaminants (HOCs) from water, effectively concentrating these substances. Also they show the potential to transfer these substances to organisms upon ingestion, although this topic is subject of debate^9–11^.

In fact, plastic pellets act as passive samplers accumulating on their surface organic pollutants present in the surrounding environment^12^. This aspect is particularly relevant in aquatic environment, where the amounts of environmental organic contaminants adsorbed on the plastic surface can be several orders of magnitude higher than that in the surrounding waters^13^. Since the first report that highlighted the presence of toxics in pellets^14^, several other studies have shown the incorporation of toxic compounds in plastic pellets, such as PCBs^4,7,15–17^, dioxin-like chemicals^11^, OCPs^4,17^, PAHs^17^ and DDTs^16^. Notably, the concentration of these chemicals varies between the different studies and the researched areas. The key factor driving this variability appears to be the time spent in water environment^16^, where pellets go through weathering and aging effects, mainly via photooxidation^2^. Due to weathering effect, plastic particles commonly undergo coloring of their surface, in a phenomenon known as “yellowing process”^18^. This color transformation potentially offers insights into the residence time in the marine environment, distinguishing aged pellets (yellowish, orange and brownish) from their pristine counterparts (white or translucent)^19^. It is therefore reasonable to hypothesize that more white and translucent pellets will be present near the sources of plastic pellets; on the contrary, moving away from the sources, yellowish and brownish pellets will dominate^20^.

The level of aging, along with the degree of erosion of plastic and the chemical properties of the pollutant, influence the sorption of pollutants; a number of studies, indeed, have noted a greater accumulation of POPs or DLCs in plastic pellets that presents greater aging and coloring^7,11,16,17^. If the aged and colored pellets present higher concentrations of organic contaminants, then these could be transferred more effectively to the aquatic biota^20^. It is possible to objectively quantify the degree of yellowing and weathering of plastic particles using the Yellowness Index (YI), which is a percentage value based on the yellowing of the sample and increases according to plastic degradation^17^.

However, the relationship between the aging process, pollutants accumulation on plastic pellets and their toxicity to aquatic biota, is still poorly understood^17^; just as it is a complex challenge to trace the life history of each pellet that stranded on the beach^21^.

**Figure 1:**
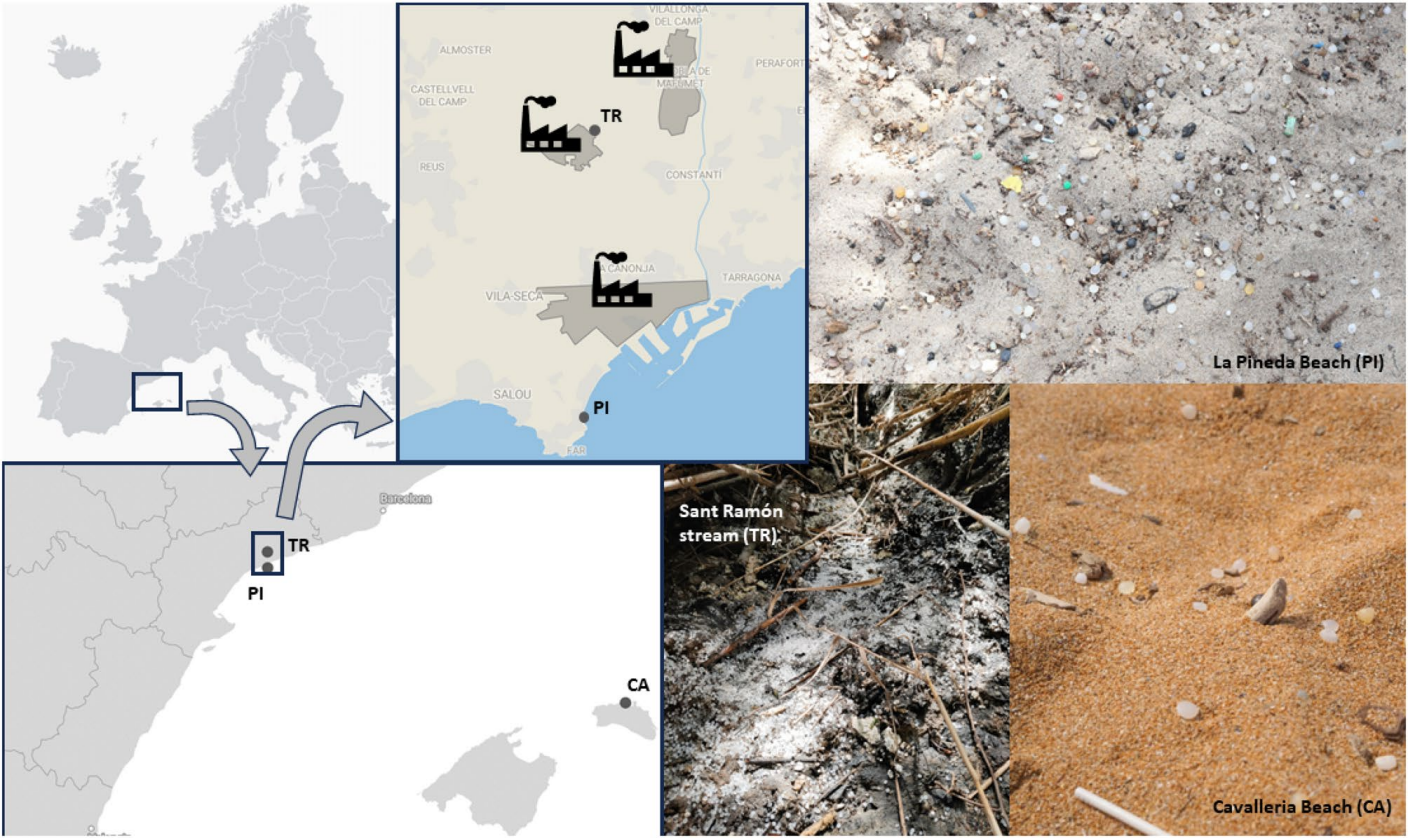
Locations where pellets were collected for this study along with photos from those locations pellet pollution. Plastic Industries are identified with industry symbol. TR Sant Ramón stream; PI Pineda Beach; CA Cavalleria Beach. Photo credits for the image of La Pineda Beach go to Anna Lofi.

The Tarragona region (Catalunya, NE Spain) is home to one of the largest and most important petrochemical complexes in the Mediterranean. This complex, known as the Chemical Complex of Tarragona (Complejo Petroquímico de Tarragona), encompasses various facilities and plants dedicated to the production, handling and logistics of chemicals and plastics (Fig. 1). The complex is strategically located near the Port of Tarragona for easy transportation of goods. Since 2018, Good Karma Projects association (goodkarmaprojects.org) has dedicated efforts to investigate and document the issue of plastic pellet pollution in Tarragona. Initial reports from witnesses highlighted occurrences of significant pellet influxes on La Pineda Beach and surrounding beaches, particularly coinciding with stormy weather or heavy rainfall in the preceding days. Initially, the prevailing belief was that these incidents were primarily linked to losses in maritime transport or originated from distant sources. However, inspections carried out in 2020 and 2021 by volunteers from our organization, in collaboration with the Corps of Rural Agents of Catalonia, within the region’s hydrographic network, have confirmed the existence of substantial pellet concentrations in these areas. Notably, these concentrations have been identified more than a dozen kilometers inland from the beach, often situated next to the facilities responsible for the production, storage, and distribution of pellets. It is widely acknowledged that companies worldwide, engaged in the handling of plastic pellets, experience unintended chronic and persistent losses^22^, and in Tarragona this is no exception.

Taking this into account, the main goal of this study is to investigate whether there are variations between plastic pellets found near their source and those collected from beaches at increasing distances from the production source, displaying signs of weathering. Our objective is to test the hypothesis that greater distances from the source lead to increased weathering and a more pronounced yellowing of the pellet samples. Additionally, we conducted toxicity assessments on leachates from these environmental samples, including sub-samples categorized by their color, using the highly sensitive sea-urchin embryo test (SET).

## Material and methods

### Sampling

Plastic pellets were collected during the spring of 2022, at three locations with increasing distance from a local source of plastic pellets waste (Fig. 1). The samples were collected as part of the Good Karma citizen science program, aiming to identify new accumulation areas in the territory. The samples were taken as evidence of the presence of pellets in these accumulation areas from a square between 50 and 100 cm wide. The sample from the Sant Ramón stream (TR, henceforth) was taken from the side of the channel where pellets accumulate among the vegetation (41° 10′ 00.5″ N 1° 11′ 08.8″ E). In the case of La Pineda Beach (PI, henceforth), samples were collected from the large southern accumulation area of the beach (41° 03′ 58.2″ N 1° 10′ 48.0″ E), about 60 m from the shoreline. Finally, in the case of Cavalleria Beach (CA, henceforth) on the island of Menorca, samples were collected in a central area of the beach, about 20 m from the shoreline, near the dunes (40° 03′ 32.9″ N 4° 04′ 32.8″ E).

### Pellets characterization

In the laboratory, plastic pellets were rinsed with distilled water to remove any attached debris. The three collected stocks were characterized according to the presence of surface cracking and their yellowness. Additional samples were sent to University of Vigo central services (CACTI) for the identification of the polymer by Fourier-Transform Infrared spectroscopy (FTIR) by using a Thermo Scientific Nicolet 6700) equipped with a attenuated total reflectance (ATR) diamond crystal.

For each stock, a representative subsample of 35 individual plastic pellets was examined by unaided visual inspection. The examination focused on the identification of two prominent indicators of photo-oxidative stress, as delineated in the classification system proposed by Hunter et al^23^. Briefly, plastic pellets were assigned weathering scores as follows: a score of ‘1’ denoted the absence of both yellowing and cracking, ‘2’ indicated the presence of either yellowing or cracking, and ‘3’ signified the coexistence of yellowing and cracking.

To calculate the yellowness index, we developed a Python script for image processing using the *SkImage* (https://scikit-image.org/)^24^, *Colour* (https://www.colour-science.org/)^25^ and *Scikit-learn* (https://scikit-learn.org) libraries^26^. The complete script is available at https://github.com/flaranjeiro/Yellowing_Index_ImageAnalysis and can be applied to any jpg image file. In summary, when you upload an image to the script, all its pixels are analyzed for color. Therefore, it is important to upload pellet images with background removed by photo editors. The software then employs statistical clustering of the color values of each pixel to identify the ten most likely colors in the image, represented in the CIE XYZ color space format. Based on this data, the Yellowness Index is computed by the Colour library, following the formula outlined in the ASTM E131 method.

Additionally, and to better understand the influence of these weathering features on the leachate toxicity, subsamples were generated for both PI and CA stations based on the degree of yellowing. These subsamples included those with no yellowing (designated as PIW and CAW), some degree of yellowing (PII and CAI), and significant yellowing (PIY and CAY). The previously described classifications were also applied on these pellet subsamples. Images of the plastic pellets samples used in this work, and analyzed by the script for Yellowness Index, can be found in supplementary material (Fig. S1).

### Toxicity tests

All pellet samples, as previously referred, were grounded with a CryoMill (Retsch) with the aid of liquid nitrogen and then sieved through a 250 µm metallic mesh to obtain a homogenous particle size. The leachate preparation followed standard methods specifically developed for plastic materials^27^. In summary, 650 mg of each sample was transferred to 65 ml glass bottles containing artificial seawater without any headspace (10 g/L). These bottles were then placed on an overhead rotator (GFL 3040) and gently rotated at 1 rpm for 24 h at a temperature of 20 °C in complete darkness. The resulting leachates were filtered through glass fiber filters (Whatman) previously cleansed with 150 ml of distilled water. Likewise, 200 ml of artificial seawater were filtered and used as control for the bioassays. To ensure optimal testing conditions, various chemical and physical parameters such as temperature, pH, salinity, and dissolved oxygen were monitored. Leachates were tested undiluted and diluted in artificial seawater to 1/3, 1/10 and 1/30.

Marine toxicity of leachates was tested using the SET bioassay according to Beiras et al^28^. Gametes were obtained by dissection of sexually mature sea urchins *Paracentrotus lividus*, collected in the outer part of the Ría de Vigo, and kept in stock at the ECIMAT (University of Vigo). Gametes viability (egg roundness and sperm motility) was assessed under the microscope and after that oocytes were transferred to a 50 ml measuring cylinder. A small volume of undiluted sperm, collected with a glass Pasteur pipette, was added to the cylinder, followed by gentle stirring with a plunger. The number of fertilized eggs, characterized by the fertilization membrane, were counted in 20 µl aliquots. Eggs with a density of 40 per ml were moved to airtight glass vials with Teflon-lined caps containing 4 ml of the treatment dilutions. In the bioassay were tested: fertilized eggs that were fixed after delivery, artificial seawater controls and dilutions of the leachates. After 48 h incubation at 20 °C in dark, samples were fixed with three drops of 30% formalin.

### Statistical analysis

The maximum length of the first 35 individuals per vials was measured using Leica image analysis software; after that, the size increase was calculated subtracting the mean egg size. Acceptability criteria in controls was fertilization > 95% and pluteus size increase > 253 µm^29^. Control corrected size increase was fit to a probit function of the leachate dilution, and the median effective concentration (EC50) was calculated as the dilution reducing size increase by 50%.

To determine the normality of the data was used the Shapiro–Wilk test, while ANOVA and Levene’s test were conducted to test differences (*p* < 0.05) among treatment group means and variances, respectively. In the event of significant differences in homoscedasticity (*p* < 0.05), Dunnett’s post hoc test was used to compare every treatment with the control treatment (filtered artificial seawater); otherwise, Dunnett’s T3 post hoc was used. Through these tests it is possible to calculate the NOEC (No Observed Effect Concentration) and the LOEC (Low Observed Effect Concentration). Finally, toxic units (TU) were calculated as TU = 1/EC20^30^. IBM SPSS statistics software V25 was used to conduct statistical analyses.

## Results

FTIR results of the randomly selected pellets from the sampled stations have shown a balanced composition of either polyethylene or polypropylene. Detailed analysis can be seen in supplementary material (Fig. S2). It is then fair to assume that both polymers were generally present in all samples and therefore polymer composition wouldn’t influence the obtained results.

**Figure 2:**
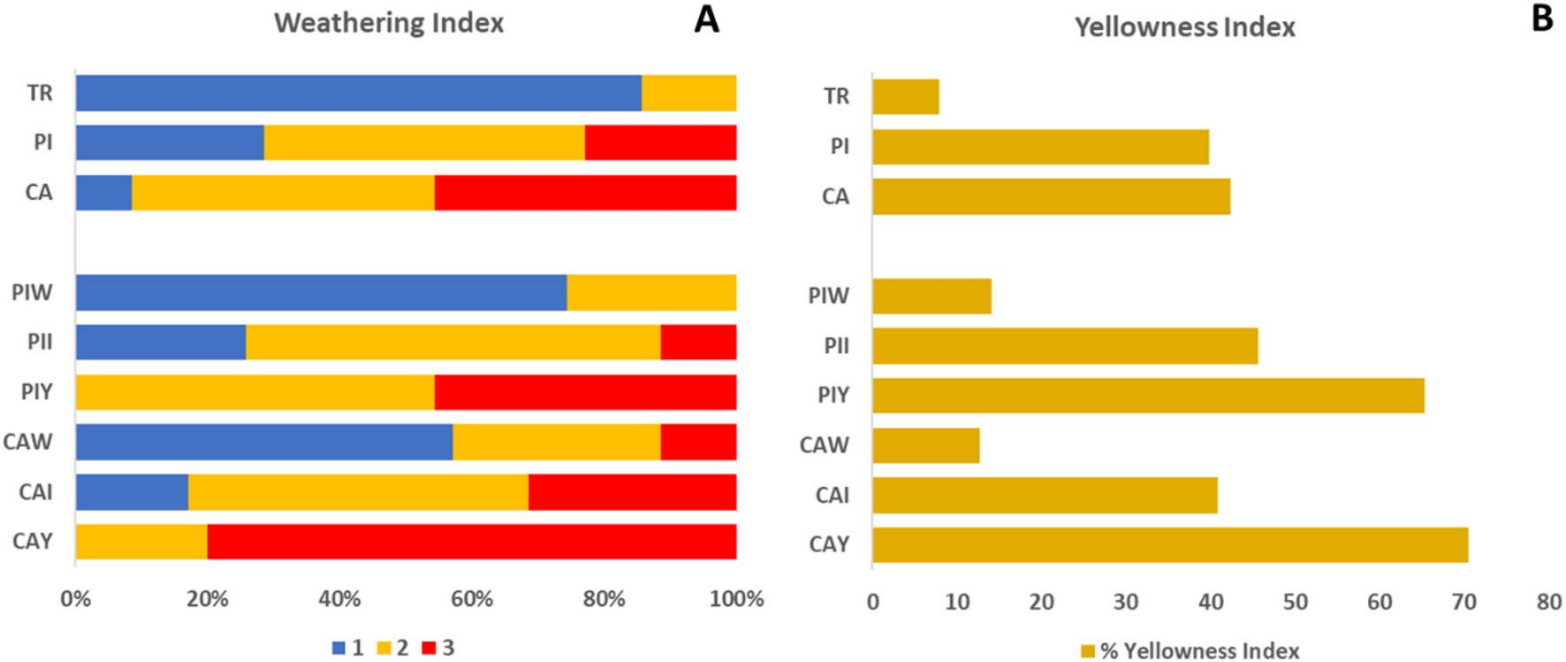
Weathering classification (**A**) and % Yellowness index (**B**) observed in this study samples and subsamples.

The categorization of visual signs of weathering reveals a consistent trend of increased weathering as one moves from inland sources to the farthest point (CA). The majority of pellets are classified as either 2 (46%) or 3 (46%) at CA, in contrast to TR, where no pellets fall into the category of 3, with the majority being classified as 1 (86%) (see Fig. 2A). PI, on the other hand, predominantly consists of pellets classified as 2 (49%). Notably, when considering subsamples selected based on visual inspection, the expected trend of increased weathering from PIW and CAW to PIY and CAY is evident. However, it is important to highlight that weathering classification is consistently higher in CA subsamples compared to their corresponding PI subsamples (eg. CAY > PIY).

As for the Yellowing classification, a similar pattern emerges (see Fig. 2B). The yellowing index generally increases from TR to CA, with less pronounced differences between PI and CA. %YI is slighlty higher in CAY compared to PIY. Interestingly, even within PI subsamples, there is a slightly higher %YI in those with no yellowing and some degree of yellowing when compared to CA. This discrepancy can be attributed to the subjective nature of visually determining color, which can vary within intermediate ranges. For a comprehensive breakdown of results and colors identified through image analysis, please refer to the Supplementary Excel file.

The outcomes of the SET, conducted using the leachates, are summarized in Table 1 (you can find dose–response curves in Fig. S3 in the supplementary material). Notably, the leachate derived from the TR sample exhibited no statistically significant toxic effects across all tested concentrations. However, significant effects were observed when using undiluted leachate from the PI sample, and in the case of the CA sample, toxicity was evident in undiluted and the three times diluted leachate.

**Table 1:**
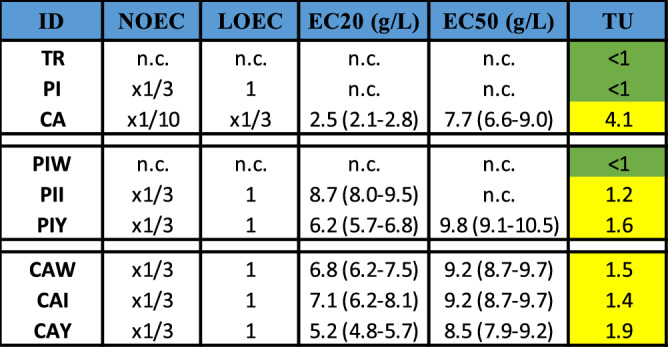
Acute toxicity data obtained for each bioassay.

n.c. = not calculable (estimation above maximum tested concentrations). For EC20 and EC50 the 95% confidence intervals are shown in parentheses.

It is worth highlighting that despite the absence of toxicity in the PI leachate as a whole, subsamples taken from PI exhibited varying toxic effects. Specifically, there were no significant effects observed in the leachates from PIW. However, both PII and PIY leachates displayed significant effects on larval growth. This time, PII and PIY were found to be slightly toxic. All CA subsamples exhibited slight toxicity, with the most heavily yellowed subsample demonstrating the highest level of toxicity (TU for CAY was 1.9, compared to 1.4 and 1.5 for CAI and CAW, respectively), mirroring the trends observed in PI. More weathered pellets from Pineda beach (PIY) result now to be more toxic than less weathered pellets subsample from Cavalleria (CAW & CAI).

**Figure 3:**
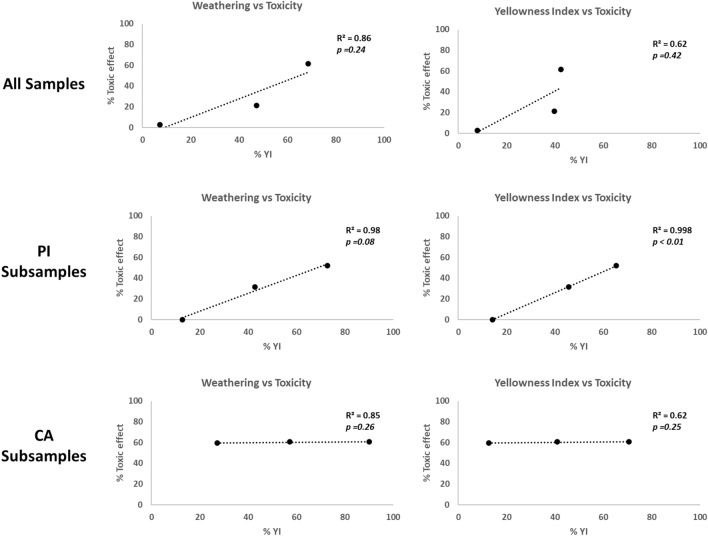
Regression analysis between weathering variables observed in pellets and toxic effects observed on sea urchin embryos.

In the regression analysis, we converted the weathering classification from Fig. 2 into percentages, with a maximum classification of 2 corresponding to 100%. The toxicity values used in the analysis were determined by calculating the average impact on larval growth (in percentage of control) observed in the undiluted leachates. Regression analysis (Fig. 3) reveals that the observed toxicity in the leachates cannot be explained by the weathering variables examined when considering all stations or when focusing solely on CA subsamples. In contrast, when looking at the PI subsamples, weathering (*p*-value < 0.1) and, to a greater extent, the Yellowing Index (*p*-value < 0.01) appear to have an influence on toxicity. However, it’s crucial to be careful when interpreting these findings due to the limited number of observations utilized in the regression analysis.

## Discussion

Plastic pellets have become a ubiquitous element of the ecosystem, been detected in numerous coastal locations worldwide^7,17,20,31,32^, freshwater ecosystems^33,34^ as well as in biota, including fish^35–37^ turtles^38–40^ and birds^41–43^. This is also the case for our study area, the Tarragona coast (Western Mediterranean), where microplastics and pellets contamination was previously reported in coastal areas^44^ and molluscs^45^.

This is a concerning issue, as plastic pellets have the potential to inflict mechanical harm on marine life by leading to complications such as intestinal blockages, reduced food consumption, and internal injuries^7,46^. But also, plastic pellets can both transport and absorb chemical compounds from their surroundings^4,47^, a process potentially influenced by the level of weathering they undergo. Some studies have reported a positive correlation between the extent of chemical contamination on the surfaces of these pellets and the degree of weathering they experience in aquatic environments^11,16,17^.

Understanding the issue of plastic pellet pollution in the Tarragona region, our research aims to provide fresh insights into the harmful effects of plastic pellets on the early life stages of marine invertebrates. Our proposed classifications confirm that plastic pellets exhibit increased weathering and yellowing as they travel farther from the source of pellet leakage. Comparing full samples there is an increase in weathering (Classification 2 + 3) of 500 and 640% from TR to PI and CA, respectively, while the yellowing increased 504% and 537% in the full sample of the same stations. In subsamples, yellowing increases up to 900% in comparison to those found in TR were registered (Fig. 2). This aligns with findings by De Monte et al.^19^, who noted that pellets left on beaches and in seawater environments for six months displayed a significant shift in color towards yellow and alterations in surface morphology. Although in our case is not possible to predict how much time pellets have been spent in the environment.

The results of SET, with the leachates of the three samples studied, show an increasing negative effect on larval growth from TR to PI and then to CA, the sample taken at the highest distance from the source which revealed to be slightly toxic. It’s important to note that PI beach is situated immediately after the riverine inflow that carries the pellets from the source to the sea, while CA is located in the Baleares Island in the midst of the Mediterranean. The hydrographic characteristics of the petrochemical complex in Tarragona designate it as a flood-prone region. Consequently, during periods of abundant rainfall, water transports particles from the soil towards the sea. Once in the sea, the pellets remain afloat on the surface, forming accumulations in proximity to their sources along the Tarragona coast. When faced with easterly or southerly winds and waves, the pellets are driven onto La Pineda Beach. Conversely, under conditions of northwesterly or westerly winds (predominant in the region), they are propelled towards the Balearic Sea. Nevertheless, and since no plastic factory is based in Balearic Islands, it’s reasonable to assume that the pellet mixture in CA includes contributions from other sources and various contaminants that may affect the overall toxicity. This could potentially account for the relatively weak correlation between the bioassay results of the sampled pellets and their weathering classifications (see Fig. 3). Nevertheless, to gain deeper insights into the influence of weathering on pellet toxicity, we took the collected samples from PI and CA and divided them into three subsamples based on the degree of pellet weathering. In the case of PI subsamples, those with no apparent weathering (PIW) exhibited no toxic effects, like what we observed in TR. However, as the level of weathering increased in the pellets, so did the negative impact on toxicity. Notably, both PII and PIY displayed slight toxicity, a phenomenon that was not observed in the assessment of the full sample. It’s worth noting that the weathering and yellowness classifications in these subsamples exhibit a strong correlation with the toxic effects of undiluted leachates (refer to Fig. 3). Likewise, the EC20 values also appear to be significantly influenced by these characteristics. As mentioned earlier, the PI sample was collected relatively close to the local source of pellet production, ensuring sample homogeneity in terms of origin. However, there exists heterogeneity in the residence time of these pellets in the environment, with those exposed for longer periods demonstrating increased toxicity. This information holds significant importance both in monitoring pellet toxicity and in managing this type of pollution. It becomes evident that even if these plastics can be considered chemically harmless at the time of production can turn toxic after an extended period in the environment. This is the case in all the CA subsamples, as they consistently displayed slight toxicity, in line with the results observed in the original CA sample. This may be attributed to the fact that, despite our attempt to categorize them into three distinct groups, all the pellets in CA had experienced a significant degree of weathering and yellowing. It’s also important to consider that this sample is likely more diverse in terms of the pellets’ sources of origin. However, even though there is no direct correlation between the toxicity of undiluted leachate and the weathering classifications (as shown in Fig. 3), both the EC50 and, notably, the EC20 demonstrate that the most weathered category is more toxic than the others.

The impact of leachates from environmental pellets on biota remains a relatively understudied and unmonitored area. Nevertheless, several studies have indicated more pronounced negative effects on embryo development when comparing beach-collected pellets to industrial polypropylene pellets in brown mussels^48^ or Polyethylene pellets in *P. lividus* larvae^49^. Similarly, research with the fish *Pimephales promelas* has shown that leachates from beached pellets are more likely to cause mortality and deformities compared to leachates from industrial polypropylene or polyethylene^50^. This can be justified by the fact that plastics have a high ability to absorb hydrophobic organic contaminants (HOCs) from water, effectively concentrating these substances^10,11^. Because of constraints related to pellet availability, we were unable to conduct chemical analyses on these samples. However, previous studies have corroborated elevated levels of toxic chemicals in weathered or aged pellets^11,17^.

These findings, alongside our results, suggest that pellets collected near the source and showing minimal weathering, are more likely to be less toxic when compared to pellets displaying significant signs of weathering and yellowing. The latter may have been exposed to the environment for an extended period, potentially leading to the absorption of chemicals from their surroundings. Nonetheless, to gain a more comprehensive understanding of this phenomenon, it will be essential to conduct additional research focused on environmental pellets and the potential toxicity of their leachates to biota. Similarly, further investigations will be necessary to scrutinize the processes of weathering and yellowing that plastic particles undergo, along with the subsequent associations between these processes and the concentration of chemical compounds.

### Supplementary Information

Supplementary Information 1.

Supplementary Information 2.

## Data Availability

The datasets used and/or analysed during the current study will be available from the corresponding author on reasonable request.
