# Supplementary material for: Weathering increases the acute toxicity of plastic pellets leachates to sea-urchin larvae—a case study with environmental samples
Source: Sci Rep. 2024 May 23;14:11784. doi: 10.1038/s41598-024-60886-x (PMC11116416; doi:10.1038/s41598-024-60886-x)
Supplement: Supplementary file 1 — Supplementary Information 1. [file 41598_2024_60886_MOESM1_ESM.docx]

Supplementary Material

Weathering Affects the Acute Toxicity of Plastic Pellets to Sea-Urchin Larvae – A Case Study with Environmental Samples

Ferrari M.^1^, Laranjeiro F.^1^, Sugrañes M.^2^, Oliva J.^2^, Beiras R.^1^

^1^ ECIMAT, Centro de Investigación Mariña (CIM), Universidade de Vigo, 36331 Vigo, Galicia, Spain

^2^Associació Good Karma Projects, Manila 49 àtic 2, 08034 Barcelona, Spain.


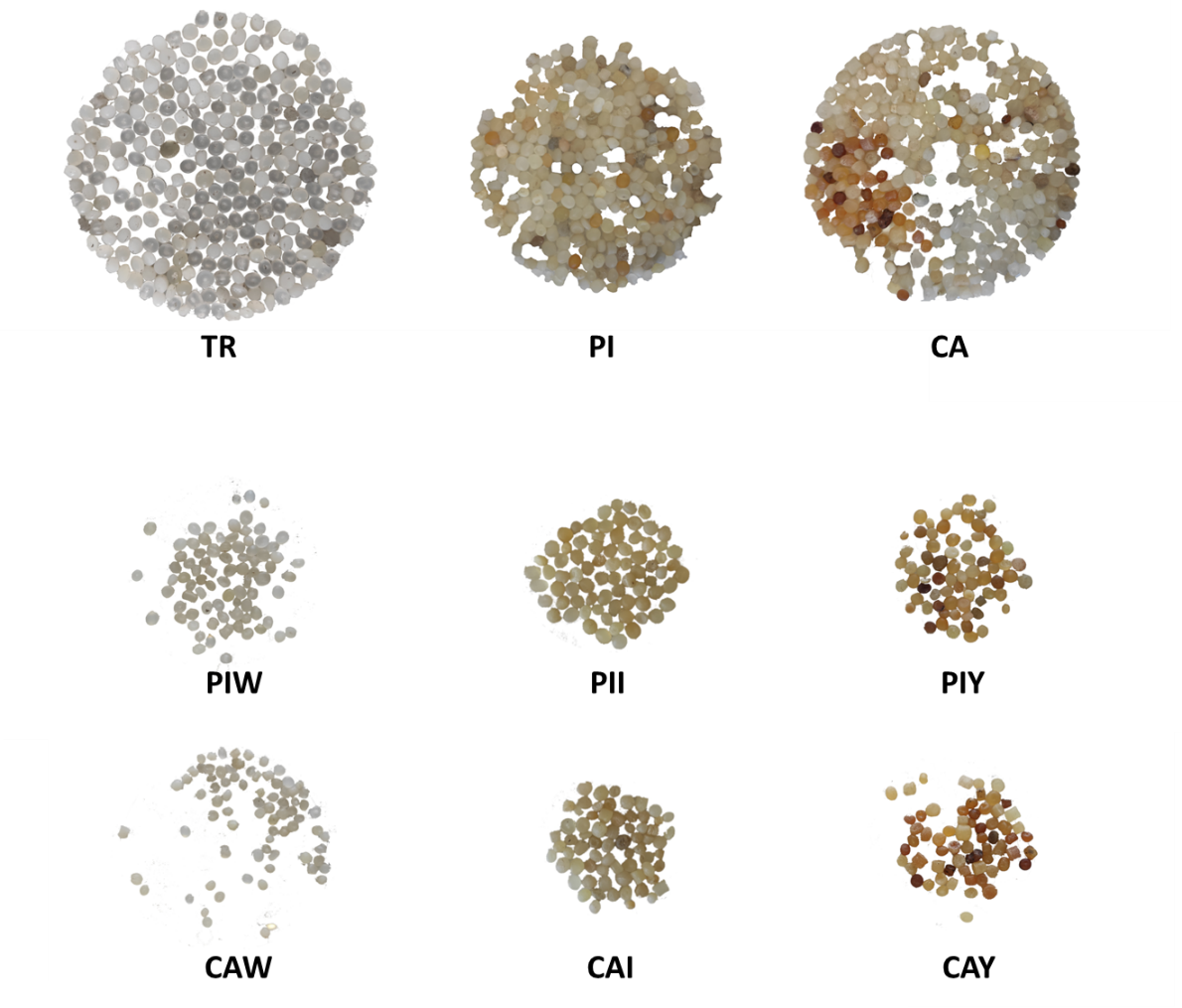


Figure S1 – Images of pellets, without background, as they were analyzed for Yellowness Index.


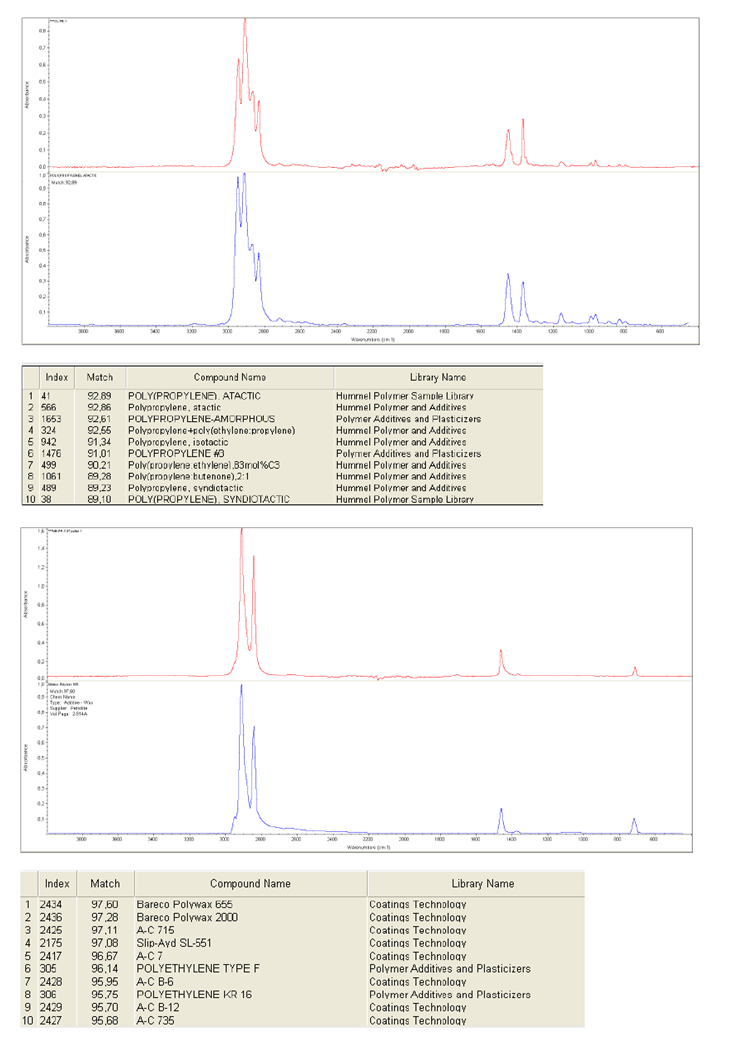


Figure S2 – FTIR spectrum of PP and PE pellets found in the samples.


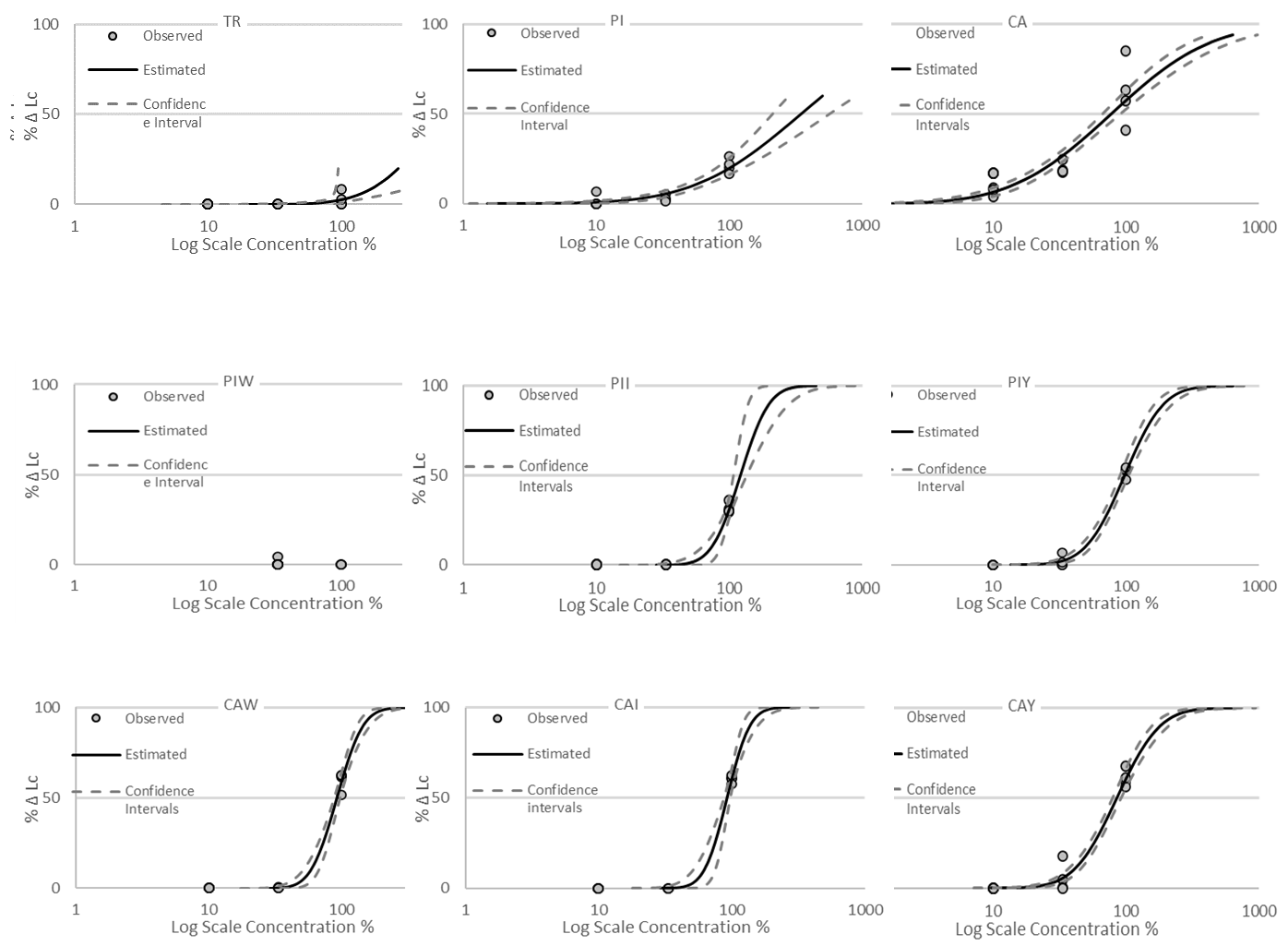


Figure S3 - Dose-response curves of tested materials. Log scale concentration is represented as percentage of leachate dilutions.
